# Heavy metal regulation of plasma membrane H+-ATPase gene expression in halophyte *Aeluropus littoralis*

**Published:** 2014-06

**Authors:** Mohsen Jam, Abbas Alemzadeh, Ali Mohammad Tale, Sara Esmaeili-Tazangi

**Affiliations:** 1 Department of Crop Production and Plant Breeding, College of Agriculture, Shiraz University, Shiraz, Iran; 2 Biotechnology Department, School of Agriculture, Shahid Bahonar University, Kerman, Iran

**Keywords:** Gouan, Lead, Mercury, Proton pump, Silver

## Abstract

The present study was conducted to find the effect of three heavy metals, Ag, Hg and Pb on the expression level of a gene encoding plasma membrane H+-ATPase in *Aeluropus littoralis*. The experiment was laid out in a completely random design with three replications. The *expression *of the main gene was normalized to the expression of the housekeeping gene actin. Two 259 and 187 bp fragments were amplified from plasma membrane H+-ATPase and actin genes using specific primers in polymerase chain reactions. The results indicated that higher concentrations of all three heavy metals declined the expression of plasma membrane H+-ATPase gene, whereas low concentrations changed the level of its transcript differently. A significant linear correlation was found between Ag concentrations of *Aeluropus littoralis *shoots and its external level; however, for Hg and Pb no correlations were observed. Root weight decreased when plants were grown at both concentrations of Ag and Hg but increased at both concentrations of Pb and NaCl. Maximum root weight was observed under lower levels of Pb, while maximum shoot weight was observed under lower levels of Hg. The greatest plant weight was obtained at low concentrations of Hg and Pb. Taken together these results show the regulation of plasma membrane H+-ATPase gene by heavy metals suggesting that *Aeluropus littoralis *can be regarded as a Phytoremediation accumulator of soils polluted with heavy metals.

## INTRODUCTION

Heavy metals, such as lead, mercury, and silver, are a group of metals which have densities greater than 5 grams per cubic centimeter [1]. The concentration of these metals in the soil varies from one area to another [2, 3]. The fate of heavy metals in soil and water depends on environmental conditions. Many factors such as ionic concentration, pH, temperature and organic or inorganic ligands affect the uptake of metals by plants [4].

Some metals such as Zn are essential while others such as Cd are not, but previousresearch shows that heavy metals are toxic at high concentrations anyway [5]. Studies have revealed that the toxicity of heavy metals can be a result of oxidative damage by the generation of reactive oxygen species (ROS) [6]. It has also been shown that heavy metals can be connected to water channel proteins, leading to the closure of stomata in the leaves of plants and stopping water flow [7].

When cells are exposed to heavy metals, plasma membrane (PM) is the first barrierto the movement of metal ions into the cytoplasm [8]. Metals have been known to cause damage to plasma membranes. Metal ions bind to sulfhydryl groups of proteins and hydroxyl groups of phospholipids [9]. They can also replace calcium ions in cell membranes and disrupt the ionic balance of the cell plasma membrane [10].

H+- ATPases are a major species of plasma membrane proteins that plays importantroles in various physiological activities of the plant like abiotic stress response. These proteins use the energy provided by ATP hydrolysis to transport protons out of the cell, generating an electrochemical gradient across the membrane [8, 11].

Previous studies indicated that the expression pattern of various genes in differentorganisms changed after exposure to heavy metals [12, 13]. Some reports also show that heavy metals differently affect gene expression patterns in plants [14, 15]. The expression level of PM H+-ATPase gene was also found to have changed under heavy metals' stress. It has been reported that the expression of a gene encoding PM H+- ATPase in cucumbers was down-regulated by cadmium treatment [16]. Nevertheless, one report showed that the expression level of proton pump did not change under the stress of heavy metals such as Cd, Cu and Ni [17].

As a rich genetic resource used for identifying new genes resistant to abiotic stress, Gouan, *Aeluropus littoralis*, is a diploid plant (2n = 2x = 16) with a relatively smallgenome of about 342 Mbp [18]. The biochemical response of *A. littoralis *to some heavymetals has been previously studied [19, 20]. Other genes that play important roles in the absorption and translocation of heavy metals have also been studied in this plant before [21]. It was shown that genes encoding PM H+-ATPase are one of the genes that have an active role in removing heavy metal ions from cytosol [22].

As of date, little research has been done to determine the expression pattern of the PM H+-ATPase gene under some heavy metal stress in some plants and to the authors' knowledge, no research has been carried out to study the expression level of this gene under Ag, Hg or Pb stress. Hence, in the present work we tried to analyze the expression pattern of a gene which encodes a PM H+-ATPase pump in *A. littoralis *and determine its relation with metal ion contents of the shoots under these heavy metals' stress.

## MATERIALS AND METHODS

**Cultivation of plants and experimental design:** *A. littoralis *seeds were collected from Maharlu Lake, south of Shiraz, surface sterilized by soaking in 1% (v/v) sodium hypochlorite for 20 min and rinsed three times with distilled water. In order to get more uniformly germinated seeds, they were placed in Petri dishes on two layers of filter paper moistened with 10 ml distilled water and incubated at 4°C for 72 h. Germinated seeds were planted in plastic pots filled with perlite and grown in a greenhouse under a16/18 h day/night cycle and 25/16°C day/night temperature. Plants were irrigated every

three days with 1/2 modified Hoagland solution [23]. After two months, the plants were treated with 1/2 modified Hoagland solution with two different concentrations (50 and

100 µl) of AgNO3, HgCl2, Pb(NO3)2 and 200 or 400 mM NaCl, separately. The experiments were carried out in a completely randomized design with 3 replicates.

**Measurement of plant heavy metal content**: Heavy metal content was measured as previously described [24]. Leaves were separated 72 h after treatment, washed and dried in an oven at 65ºC for 72 h and weighed. After drying, one gram of each sample was placed into a porcelain crucible and heated in a furnace. The furnace temperature slowly increased from room temperature to 550ºC in 1 h. Samples were ashed for 3 h. The residue was dissolved in 5 ml HCl (2 N) and the total volume was adjusted to 50 ml by adding distilled water. The metal content was then analyzed by atomic absorption spectroscopy.

**RNA extraction, preparing cDNAs: **Leaves were sampled 0, 6, 48 and 72 h after treatment, frozen in liquid nitrogen and stored at -80°C until used for RNA extraction. Total RNA was extracted from leaves using GF-1 Total RNA Extraction Kit (Vivantis, Malaysia). The quality of the extracted RNA was assessed by electrophoresis on 1% agarose gel. First-strand cDNAs were prepared from 1 µg total RNA by RevertAid M- MuLV reverse transcriptase (Fermentas, Germany) with oligo (dt) primer.

**PCR reactions: **The expression pattern of PM H+-ATPase gene was investigated in the leaves by semiquantitative RT-PCR. The cDNAs were amplified by specific primers (Table 1) for genes, encoding PM H+-ATPase as the target and the actin as reference genes. PCR reactions in a final volume of 25 µl reaction mixture containing 10 mM tris- HCl (pH 8.3), 50 mM KCl, 1.5 MgCl2, 200 µM dNTPs, 1 µl diluted cDNAs, 0.3 µM of each primer and 1 unit taq DNA polymerase were carried out under the following conditions: 5 min at 95 °C, followed by 28 one minute cycles at 95°C, 30 s at 60°C, and1 min at 72°C, with a final extension step of 7 min at 72°C. The PCR product was then separated on 1% agarose gel.

**Statistical analysis: **The experiments were repeated three times. Data in tables and figures represent mean values. All data were analyzed by analysis of variance (ANOVA) procedures using SAS (version 9.3). Treatment means were separated by Duncan's multiple range test (P<0.05).

## RESULTS AND DISCUSSION

The effects of different concentrations of various heavy metals and NaCl on shoot and root weight are shown in Table 2. The results showed that heavy metals differently affected the weight of the plants. Root weight increased under Pb and NaCl treatments as compared to the control and the increment was greater in low levels, but Hg and Ag had no significant effect. Maximum weight was observed under lower levels of Pb (50µM) which could probably be due to the immobilization of lead and its accumulation inroots. Reduced root weight was noticed when plants were grown at high concentrations of Pb (Table 2). This could be related to the potentially devastating effect of high concentrations of Pb (100 µM) on plant cells which leads to the reduction of the roots' growth rate. Pb has also been reported to have a major effect on membrane stability in safflower [25]. Reductions of root growth rate with increasing external Pb supply levels in other plants have also been reported [26].

**Table 1:** The sequences of primers used to amplify the genes encoding a plasma membrane H^+^-ATPase (target gene) and actin as reference gene in PCR

**Genes**	**Name of Primer**	**Sequence**	**TM (** **°** **C)**
Target gene	PMH1RTPF	5'- ACCTGAGAAGACCAAGGAGTCT-3'	62.15
	PMH1RTPR	5'- TACAGGAAGTGCTTCAAGTGTAG-3'	60.00
Actin gene	ActinAlF	5'- CGTACAACTCCATCATGAAGTG-3'	61.96
	ActinAlR	5'- CAAACACTGTACTTTCTCTCCG-3'	60.35

**Table 2:** The weight of shoot and root of *A.* *littoralis* plants under different heavy metals and NaCl treatments

**Treatment (mM)**	**Root weight (g)**	**Shoot weight (g)**	**Plant weight ((g)**
Control	0.011d	0.060bc	0.071bcd
Ag (0.05)	0.007de	0.047cd	0.054de
Ag (0.10)	0.005e	0.033d	0.038 e
Hg (0.05)	0.009de	0.122a	0.131a
Hg (0.10)	0.007de	0.058bc	0.065 cd
Pb (0.05)	0.049a	0.075b	0.124a
Pb (0.10)	0.022c	0.049cd	0.071bcd
NaCl (200)	0.028b	0.060bc	0.088 b
NaCl (400)	0.024c	0.058bc	0.082bc

As compared with the control, the increase in shoot fresh weight was statistically significant at an Hg supply level of 50 µM; however, no significant increment was observed in other treatments even at high levels of Hg (Table 2). In response to Ag treatment, shoot and root weights decreased as compared with the control and this decrease was significant at high Ag concentrations (Table 2). These results are consistent with those of Brandt *et al*. (2005) who showed that lettuce growth was negatively affected by silver [27]. NaCl treatment increased the root weight in both levels, the increment being greater at the high level (400 mM). NaCl is found to have increased plant weight in halophytic plants such as *Salvadora persica *[28]. It has been showed that the weight of halophyte *Cynara cardunculus *roots increased for plants growing in NaCl but not for those grown in KCl [29]. This indicates that high external NaCl is less toxic than KCl for halophytic plants. They also reported that the deleterious effect of salts on growth was more evident in shoots than roots. Our results also show that NaCl did not increase the weight of shoots in *A. littoralis, *although it significantly increased the weight of roots in both levels (Table 2).

The heavy metals' contents of shoots in the plants treated with Pb, Ag and Hgsignificantly increased, as compared with the control. Generally, the metal concentration in the shoot of the plants under different treatments was in the descending order of Ag>Pb>Hg. The Ag content in the shoot of *A. littoralis *increased with increasing external Ag supply levels, whereas no difference was found between the two supply levels of Hg and Pb (Table 3). Ag concentration increased in the shoot of Ag- treated plants by more than seven times in comparison to the control. A significant linear correlation was found between the Ag concentrations in the shoots of *A. littoralis *and the external Ag supply level (Table 3).

RNA quality was assessed by electrophoresis on 1% agarose gel. TheOD260/OD280 ratio of extracted RNA was 1.9 and the bands corresponding to 18S and

**Figure 1:**
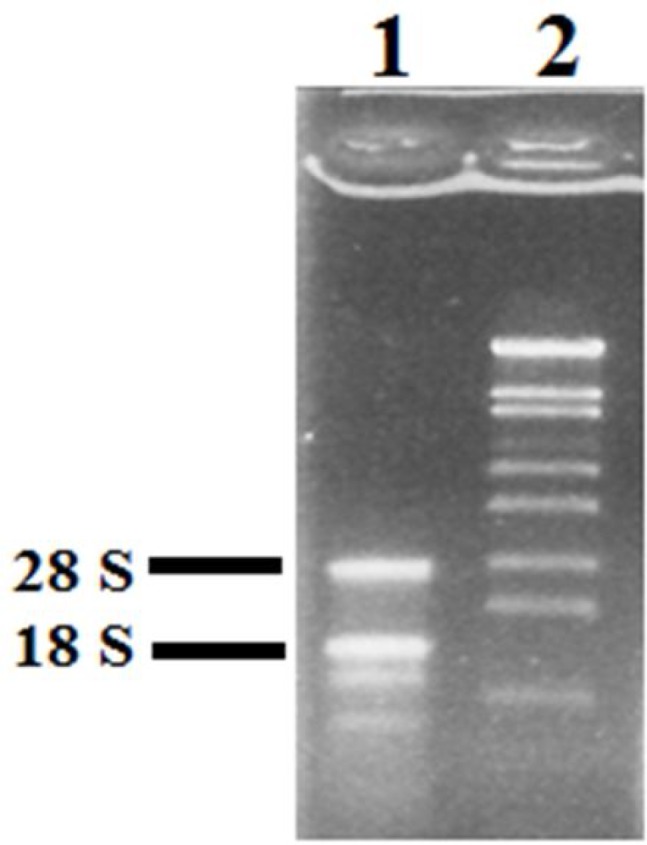
Total RNA extracted from leaves of *A.* *littoralis* was separated by 1% agarose gel electrophoresis and stained with ethidium bromide. 1) Total RNA extracted from sample and 2) size marker λ/*Sty*I

28S rRNA were distinctly visible on the gel, indicating high quality and non-degradedRNA (Fig. 1).

RT-PCR is a powerful technique to evaluate the quality of RNA samples, because it is sensitive to the degradation of RNA or to the presence of inhibitors in the reaction. Hence, cDNAs prepared from RNA were used as template in a PCR reaction to test RNA quality. Two fragments with lengths of 187 and 259 bp were amplified with specific primers for actin and PM H+-ATPase genes respectively, which indicated the high quality of the prepared cDNAs.

**Table 3:** Contents of Ag, Hg and Pb in shoot of *Aeluropus* *littoralis *exposed to different concentrations of heavy metals.

**He levels** **Control**	**Hg uptake** **(mg** ** kg** ^-1^ **)** **38** ^b^	**Ag levels** **Control**	**Ag uptake** **(mg** ** kg** ^-1^ **)** **610** ^c^	**Pb levels** **Control**	**Pb uptake** **(mg kg** ^-1^ **)** **48** ^b^
50 (μM)	60^a^	50 (μM)	2290^b^	50 (μM)	122^a^
100 (μM)	68^a^	100 (μM)	4330^a^	100 (μM)	126^a^

**Figure 2:**
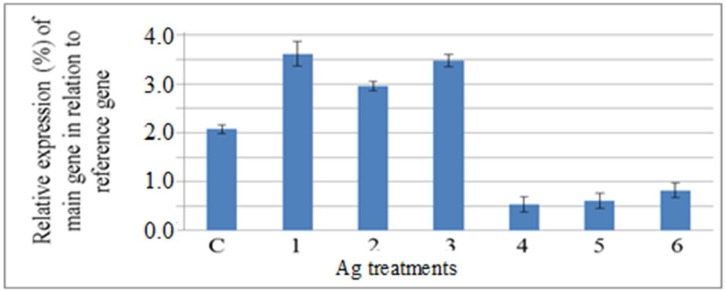
Semiquantitative analysis of the expression level of plasma membrane H^+^-ATPase gene, main gene, in the shoot of A. *littoralis* exposed to Ag (normalized by the level of actin gene, as a reference gene). 1, 2 and 3) samples were harvested from plants treated with 50 μM Ag after 6, 48 and 72 h, respectively. 4, 5 and 6) samples were harvested from plants treated with 100 μM Ag after 6, 48 and 72 h, respectively. C) Sample was harvested from control plants

**Figure 3:**
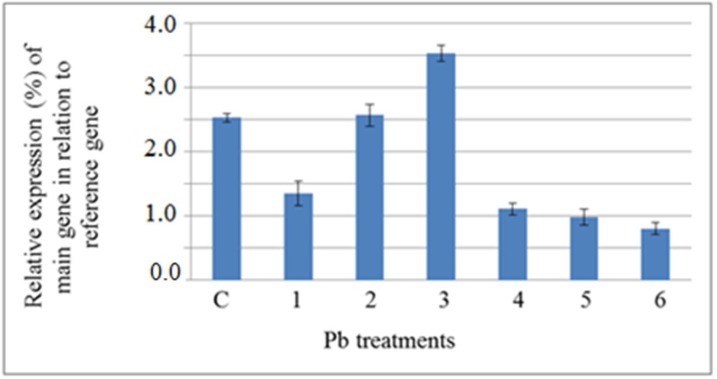
Semiquantitative analysis of the expression level of plasma membrane H+-ATPase gene, main gene, in the shoot of *A.* *littoralis* exposed to Pb (normalized by the level of actin gene, as a reference gene). 1, 2 and 3) samples were harvested from plants treated with 50 µM Pb after 6, 48 and 72 h, respectively. 4, 5 and 6) samples were harvested from plants treated with 100 µM Pb after 6, 48 and 72 h, respectively. C) Sample was harvested from control plants

**Figure 4:**
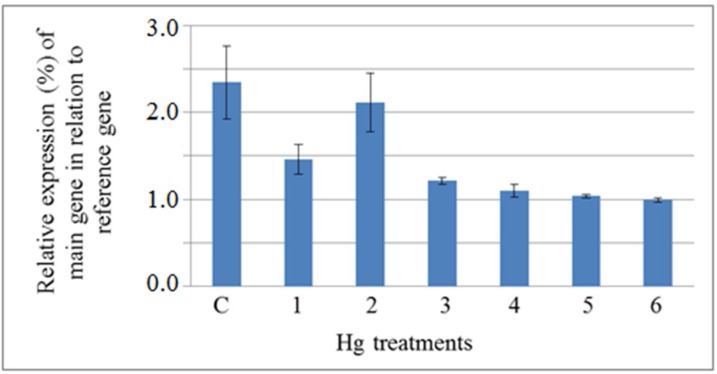
Semiquantitative analysis of the expression level of plasma membrane H+-ATPase gene, main gene, in the shoot of *A.* *littoralis* exposed to Hg (normalized by the level of actin gene, as a reference gene). 1, 2 and 3) samples were harvested from plants treated with 50 µM Hg after 6, 48 and 72 h, respectively. 4, 5 and 6) samples were harvested from plants treated with 100 µM Hg after 6, 48 and 72 h, respectively. C) Sample was harvested from control plants

**Figure 5:**
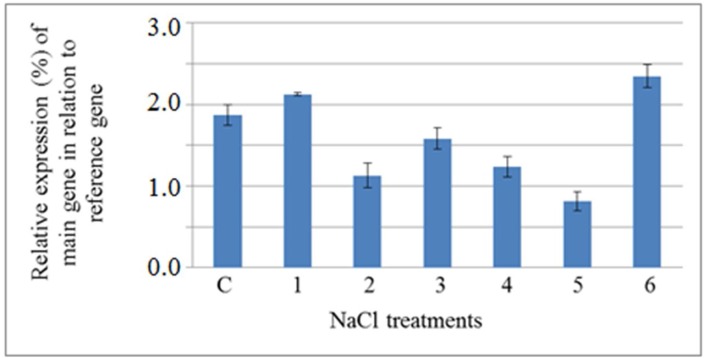
Semiquantitative analysis of the expression level of plasma membrane H+-ATPase gene, main gene, in the shoot of *A.* *littoralis* exposed to NaCl (normalized by the level of actin gene, as a reference gene). 1, 2 and 3) samples were harvested from plants treated with 200 mM NaCl after 6, 48 and 72 h, respectively. 4, 5 and 6) samples were harvested from plants treated with 400 µM NaCl after 6, 48 and 72 h, respectively. C) Sample was harvested from control plants.

Treatment of gouan plants with heavy metals (Ag, Hg and Pb) significantly affected the expression levels of PM H+-ATPase. The effect of high concentrations of Ag, Hg and Pb was similar, and all heavy metals in high external supply levels decreased the expression level of PM H+-ATPase (Fig. 2-4), whereas a high level of NaCl(400 mM) increased the expression level of this gene after 72 h (Fig. 5). Our results are consistent with those of Janicka- Russak et al. (2008) who reported that high concentrations (100µM) of Cd decreased the expression of *CsHA3*, a gene encoding a PM H+-ATPase in

*Cucumis sativus*, whereas the transcript level of the *CsHA3 *gene in plants treated withlow concentrations of Cd (10 µM) was similar to the control [16].

The expressions of the PM H+-ATPase gene in plants treated with low concentrations (50 µM) of all three heavy metals were significantly higher than plantstreated with high concentrations. Low concentrations of Ag caused the significant increment of PM H+-ATPase gene expression after 6, 48 or 72 h (Fig. 2). On the other hand, plant weight significantly decreased in plants treated with low concentrations (50 µM) of Ag in comparison to the control (Table 3). In addition, Ag concentration increased in the shoots of plants treated with Ag more than other heavy metals. It can be suggested that Ag stimulated the expression of the PM H+-ATPase gene under low concentrations of external Ag supply, but higher concentrations caused the distinct inhibition of the PM H+-ATPase transcript.

In low concentrations, the accumulation of PM H+-ATPase gene transcript in Pb- treated plants decreased and reached a minimum at 6 h but increased afterwards from 6 h to 72 h (Fig. 3). It has been previously reported that the expression of some genes increased in response to Pb treatments in *Arabidopsis thaliana *[30]. These studies showed that some gene encoding enzymes that were involved in sulfur assimilation, GSH metabolism, indol-3-acetic acid and jasmonic acid biosynthesis were up-regulated, and that these pathways were linked, through signaling transduction, to biosynthesis metal detoxification and transport molecules [30]. Moreover, it has been shown that the expression of *AtPDR12*, a gene encoding an ABC transporter in *A. thaliana*, increased in response to Pb treatment and that AtPDR12 contributes to the resistance ofArabidopsis to Pb [31]. It can be suggested that like ABC transporters, PM H+-ATPase contributes to the resistance of plants to Pb by pumping or regulating the transport of Pbor Pb-related toxic compounds to the exterior of the cell.

In the case of Hg, the expression level of PM H+-ATPase decreased in the shoots for a short time, increased after 48 h, and decreased again after 72 h (Fig. 4). It has beenpreviously reported that the levels of RNA, DNA and protein were affected in HgCl2treated cells [32], while the effect of Hg on gene expression in plant cells has not been

reported yet.

Although many studies have shown changes in the gene expression of PM H+- ATPase in response to a variety of environmental factors, including drought, salt andmechanical stress [33, 34], to our knowledge, data concerning the effect of heavy metals on the PM H+-ATPase expression is very limited and no study has yet reported the effect of Ag, Hg or Pb on the gene expression of this pump.

Taken together, the results showed that with the exception of Pb and Hg at low concentrations, heavy metals reduced the weight of plants, while NaCl increased fresh weight at both concentrations. The shoots of gouan did not accumulate heavy metals with similar efficiency. A significant linear correlation was found between the concentration of Ag in the shoots of *A. littoralis *and that of the external supply level, while in the case of Hg and Pb no correlation was observed. The expression level of the PM H+-ATPase gene was changed by 50 µM Ag, Hg and Pb but decreased with high levels of these heavy metals.

The results indicate that *A. littoralis *can accumulate exceptional concentrations of Pb in its root without showing toxicity symptoms. Plant species, belonging to the accumulator class, have exhibited higher tolerance to excessive levels of Pb in the growth media [35]. Hence, *A. littoralis *may be introduced as an accumulator for this metal.
